# *Cistanche* extracts ameliorates the neurotoxicity induced by hydrogen peroxide in new mutant *DJ‐1*‐transfected neuroblastoma cellular models

**DOI:** 10.1002/brb3.1304

**Published:** 2019-06-19

**Authors:** Chunna An, Xiaoping Pu, Qi Wang, Hongning Zhang

**Affiliations:** ^1^ Department of Pharmacology School of Basic Medical Sciences North China University of Science and Technology Tangshan China; ^2^ Department of Molecular and Cellular Pharmacology School of Pharmaceutical Sciences Peking University Beijing China; ^3^ Department of Pharmacology School of Basic Medical Sciences Capital Medical University Beijing China

**Keywords:** *Cistanche* extracts, mutant *DJ‐1*, neuroblastoma cells, Parkinson's disease

## Abstract

**Introduction:**

DJ‐1 mutation is a causative reason for familial Parkinson's disease (PD). Leucine166Proline (L166P) and C106S are two important DJ‐1 mutations. In this study, we established hydrogen peroxide (H_2_O_2_) induced L166P and C106S DJ‐1‐transfected neuroblastoma (SH‐SY5Y) cellular models of PD and investigated the effects of *Cistanche* extracts and key bioactive compounds, including acteoside, echinacoside, caffeic acid, and *Cistanche* total glycosides on these two models.

**Methods:**

After expressing FLAG‐tagged L166P and C106S DJ‐1 plasmids in *Escherichia coli*, the expressed plasmids were collected, treated with restriction enzyme, and identified using DNA electrophoresis. After purification, the L166P DJ‐1 and C106S DJ‐1 plasmids were separately transfected into SH‐SY5Y cells using liposomes. Transfected SH‐SY5Y cells were detected by western blotting and immunocytochemistry. Cell viability was determined using MTT assay.

**Results:**

Both western blotting and immunocytochemistry showed that L166P and C106S DJ‐1 were highly expressed in the transfected SH‐SY5Y cells. MTT assays showed that transfection with L166P or C106S DJ‐1 reduced the viability of SH‐SY5Y cells exposed to H_2_O_2_, as compared to untransfected SH‐SY5Y cells. In addition, *Cistanche* extracts and key bioactive compounds, including acteoside, echinacoside, caffeic acid, and *Cistanche* total glycosides, significantly inhibited the decreases of cell viability caused by H_2_O_2_ in L166P and C106S DJ‐1‐transfected SH‐SY5Y cells.

**Conclusions:**

These findings suggest that we successfully established sensitive and stable H_2_O_2_ induced L166P DJ‐1‐ and C106S DJ‐1‐transfected SH‐SY5Y cell models of PD and *Cistanche* extracts may thus be useful for treating PD.

## 1 INTRODUCTION

The main neurodegenerative diseases include Alzheimer's disease, Parkinson's disease (PD), Huntington's disease, and others. Among these disorders, the incidence rate of PD is second in the world and achieves to 1%–2% in the people over 60 years old (Burke & O'Malley, 2013; Mullard, 2017; Shen & Ji, 2013). As the major neurological characteristics of PD, the dopaminergic neurons in the substantia nigra pars compacta is progressive degenerative (Glizer & MacDonald, 2016), which is accompanied by the appearance of α‐synuclein inclusions called Lewy bodies (Zhang, An, Zhang, & Pu, 2010). In the pathogenesis of PD, rest tremors, rigidity, bradykinesia, and postural abnormalities are be diagnosed as main symptoms of PD in clinic. Although the exact progressive degenerative mechanisms of dopaminergic neurons are not understandable, either environmental causes including exposure to insecticides, neurotoxic agents, and heavy metals, or genetic causes such as mutations of *Parkin* (Kitada et al., 1998), α*‐synuclein* (Polymeropoulos et al., 1997), and *DJ‐1* (Biosa et al., 2017; Bonifati et al., 2003), are believed to leading to the occurrence of PD. To date, the numbers of missense mutations, frameshift mutations and large fragment deletions caused *DJ‐1* gene mutations exceed to 10 (Andres‐Mateos et al., 2007; Bonifati et al., 2003; Hague et al., 2003). Within DJ‐1 protein, the substitution of Leucine166Proline (L166P) stands out as important. In addition, it has also been shown that oxidative stress occurs in PD (Dexter et al., 1989, 1994; Nikam, Nikam, Ahaley, & Sontakke, 2009) and is involved in its pathogenesis (Hague et al., 2003).

*DJ‐1* gene containing eight exons distributed over 24 kb, locates on chromosome 1p36 in humans (Bonifati et al., 2003), and encodes a highly conserved protein containing 189 amino acids. The protein belonged to the ThiJ/PfpI family, exists in a homodimer form. Glial cells within the cortex and substantia nigra and striatum are the main found in areas of DJ‐1 protein (Olzmann et al., 2007). In these cells, DJ‐1 plays the following roles, such as oncogene (Nagakubo et al., 1997), transcriptional regulation (Kim et al., 2005; Niki, Takahashi‐Niki, Taira, Iguchi‐Ariga, & Agria, 2003), antioxidative stress (Taira et al., 2004; Zhou & Freed, 2005), chaperone (Shendelman, Jonason, Marlinat, Leete, & Abeliovich, 2004; Zhou, Zhu, Wioson, Petsko, & Fink, 2006), and protease (Abou‐Sleiman, Healy, Quinn, Lees, & Wood, 2003).

*Cistanche* is a traditional Chinese medicine, and has been mainly used to treat andrology disease down the ages. The major active ingredients of *Cistanche* are phenylglycosides including 34 compounds, such as acteoside, echinacoside, etc. The pharmacological effects of *Cistanche* extracts include improving sexual function, antiaging, increasing learning and memory ability, neuroprotection, immunomodulation, antifatigue, antiischemic, and liver protection (Tu et al., 2011). Caffeic acid is one of major metabolites of *Cistanche* (Yan, 2018).

The commonly used cellular models of PD include 1‐methyl‐4‐phenylpyridinium ion‐induced PC12 cells (Abou‐Sleiman et al., 2003) and hydrogen peroxide (H_2_O_2_)‐induced neuroblastoma (SH‐SY5Y) cells (Zhang et al., 2009). However, the typical pathological features of PD are not present in these models. To develop a more physiologically relevant cellular model of PD, in this study we established H_2_O_2_ induced L166P and C106S DJ‐1‐transfected SH‐SY5Y cells, and investigated the effects of *Cistanche* extracts and key bioactive compounds, including acteoside, echinacoside, caffeic acid, and *Cistanche* total glycosides on these two models, for the first time.

## 2 MATERIALS AND METHODS

### 2.1 Plasmids, drugs, chemicals, and cells

FLAG‐L166P DJ‐1, FLAG‐C106S DJ‐1 plasmids, and anti‐DJ‐1 polyclonal antibody were kindly supplied by Dr. Hiroyoshi Ariga, Graduate School of Pharmaceutical Sciences, Hokkaido University. Dehydrated minimal essential medium (MEM) and F‐12 medium were purchased from Gibco. Lipofectin was from Invitrogen. FastDigest Xho I and FastDigest EcoR I were from Fermentas. Endotoxin‐free plasmid preparation kit was from BioTeke. The SH‐SY5Y cell line was from the Cell Bank of the Chinese Academy of Medical Sciences. BCA protein assay reagent kit was from Pierce. PVDF membranes were from Millipore. Anti‐FLAG polyclonal antibody was from GeneTex. *Cistanche* extracts, including acteoside, echinacoside, caffeic acid, and *Cistanche* total glycosides, were supplied by the Department of Natural Medicines, School of Pharmaceutical Sciences, Peking University.

### 2.2 Plasmids amplification, extraction, and purification

After culturing *Escherichia coli* JM109 cells in LB liquid medium until OD_600_ = 0.5, competent cells were prepared using the calcium chloride method. The competent *E. coli* were then transformed with plasmid DNA using the heat shock method, after which the transformants were cultured on LB plates containing ampicillin for 16–24 hr at 37°C. A successful transformed monoclonal colony was then selected and cultured in LB liquid medium containing 50 μg/mL ampicillin for an additional 12 hr at 37°C. The plasmids were extracted and purified using an endotoxin‐free plasmid preparation kit according to the manufacturer's instructions.

### 2.3 Plasmid identification

Extracted plasmids were subjected to enzymatic digestion, after which the plasmids and digested fragments were examined using 1% agarose gel electrophoresis. The resultant gel was stained in EB solution for 20–25 min at room temperature and photographed.

### 2.4 Plasmid transfection into SH‐SY5Y cells

Purified plasmid DNA was transfected into SH‐SY5Y cells cultured in MEM/F‐12 medium containing 10% fetal bovine serum. Transfected cells were then selected in medium containing 400 μg/mL G418, after which 200 μg/mL G418 was used to maintain the stably transfected cell line.

### 2.5 Identification of transfected cells by Western blot

Transfected SH‐SY5Y cells were lysed in RIPA buffer. After centrifugation of the lysate, the total protein concentration in the supernatant was determined using a BCA protein assay reagent kit. Equal amounts of protein extract were then subjected to a 12.5% SDS‐polyacrylamide gel electrophoresis and transferred onto a PVDF membrane. The PVDF membrane was blocked with 5% nonfat dry milk in TBST solution and processed for immunodetection. Anti‐FLAG and anti‐DJ‐1 were used as primary antibodies, and HRP‐conjugated IgG was the secondary antibody. An enhanced chemiluminescence detection system was applied to detect the target proteins.

### 2.6 Immunocytochemical identification of transfected cells

Transfected SH‐SY5Y cells were fixed with 4% paraformaldehyde solution, permeabilized with 0.3% triton X‐100 solution and blocked with 10% goat serum. The blocked cells were then incubated first with anti‐FLAG and anti‐DJ‐1 polyclonal antibody, and then with FITC‐conjugated goat antirabbit secondary antibody. The cell nuclei were stained with Hoechst 33342, and the cells were visualized under an inverted fluorescence microscope (IX‐71, Olympus).

### 2.7 Cell viability assay

Untransfected and transfected SH‐SY5Y cells were plated into a 96‐well plate at a density of 5 × 10^4^ cells/well, incubated for 24 hr, and treated with graded H_2_O_2_ (0.1, 0.2, 0.3, 0.4, 0.5, and 1 mM, respectively) for 1 hr. The medium was then discarded, and 0.5 g/L MTT was added. After incubation for 4 hr, the medium was discarded, and 200 μL of DMSO was added to dissolve the formazan formed by the viable cells. The numbers of viable cells were then estimated based on the OD_570_ of the solution.

### 2.8 Effects of *Cistanche* extracts on cell viability

Transfected cells were plated into a 96‐well plate at a density of 5 × 10^4^ cell/well and incubated for 24 hr. Thereafter, *Cistanche* extracts, including acteoside, echinacoside, caffeic acid, and *Cistanche* total glycosides (all 10, 20, and 40 μg/mL, respectively), were added and the cells were incubated for 6 hr before treatment with 0.2 mM H_2_O_2_ for an additional 1 hr. The medium was the discarded, and cell viability was assayed using MTT as described above.

### 2.9 Statistical analysis

Data are expressed as the mean ± *SD* of three independent experiments. Differences between groups were analyzed using one‐way ANOVA and the LSD method with SPSS 22.0 software. Values of *p *<* *0.05 were considered significant.

## 3 RESULTS

### 3.1 Plasmids were successfully transformed and purified

**Figure 1:**
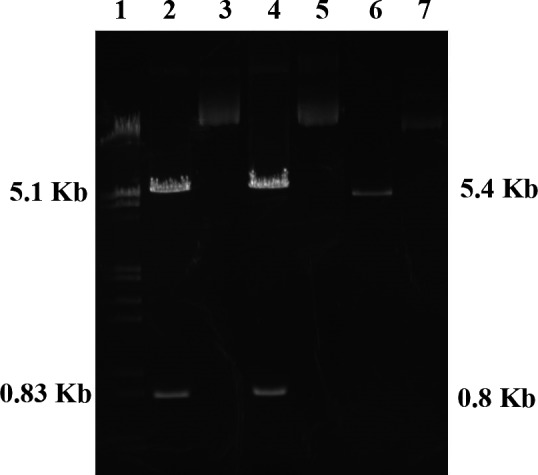
Results of restriction enzymolysis and agarose electrophoresis of L166P and C106S DJ‐1 plasmid. 1: marker; 2: extracted L166P DJ‐1 plasmid treated with restriction enzymes; 3: extracted L166P DJ‐1 plasmid; 4: extracted C106S DJ‐1 plasmid treated with restriction enzymes; 5: extracted C106S DJ‐1 plasmid; 6. plasmid treated with restriction enzymes; 7. plasmid

After separate expression in *E. coli* JM109, plasmids extracted and examined using 1% agarose gel electrophoresis after digestion with a restriction enzyme (Figure 1). The 6.2‐kb plasmids were cleaved into 5.4‐ and 0.8‐kb linear DNA fragments, which confirmed that the L166P and C106S DJ‐1 plasmids were both successfully expressed and purified.

### 3.2 Identification of transfected cells by Western blotting

**Figure 2:**
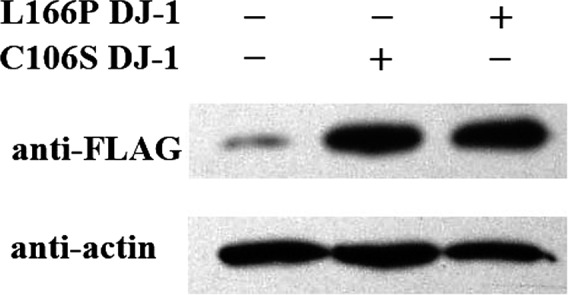
Western blot analysis

As shown in Figure 2, we detected FLAG‐L166P DJ‐1 and FLAG‐C106S DJ‐1 bands at about 70 kDa. The high levels of FLAG‐tagged protein in the transfected SH‐SY5Y cells indicate that the L166P and C106S DJ‐1 mutants were strongly expressed in their respective transfectants.

### 3.3 Immunocytochemical identification of transfected cells

**Figure 3:**
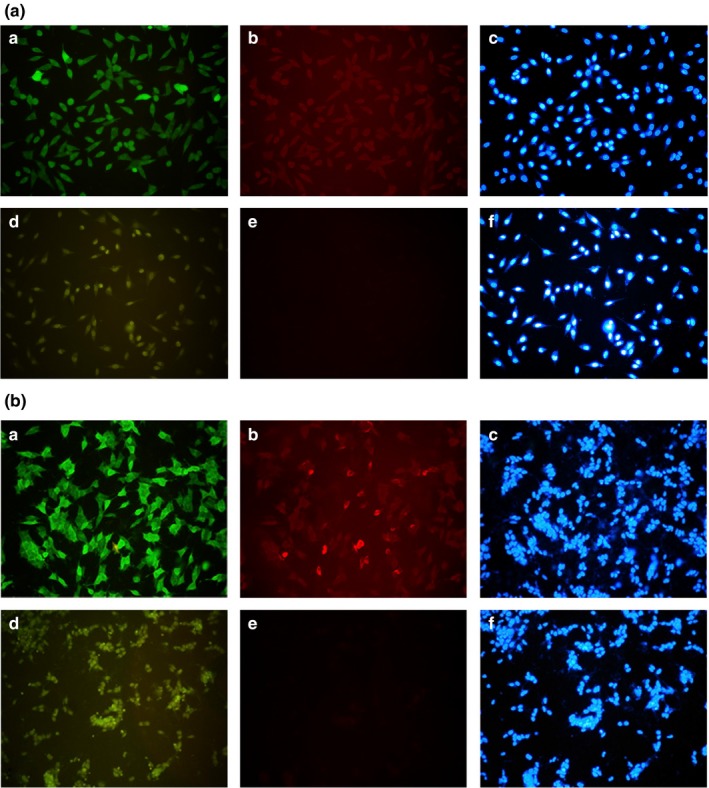
Immunocytochemical analysis of SH‐SY5Y cells transfected with L166P DJ‐1 (A) or C106S DJ‐1 (B) (200×). a, b, c: Positive staining with anti‐DJ‐1 antibody, anti‐FLAG antibody and Hoechst 33342. d, e, f: Negative controls

Figure 3 shows the strong fluorescent signals from transfected SH‐SY5Y cells after incubation with anti‐DJ‐1 or anti‐FLAG antibody. This confirms the high levels of L166P and C106S DJ‐1 expressed in the transfectants.

### 3.4 Transfected cells were more sensitive to H_2_O_2_ than untransfected cells

After incubation for 1 hr in the presence of graded concentrations of H_2_O_2_ (0.1, 0.2, 0.3, 0.4, 0.5, and 1 mM, respectively), the viabilities of SH‐SY5Y cells transfected with L166P or C106S DJ‐1 were dose‐dependently reduced as compared to untransfected cells (Table 1).

**Table 1:** The effect of H_2_O_2_ on cell viability ($\bar{x}$±S, *n* = 3)

Groups	Concentration of H_2_O_2_ (mM)						
	0	0.1	0.2	0.3	0.4	0.5	1.0
SH‐SY5Y	100 ± 0	95.4 ± 2.9	85.1 ± 6.1	52.7 ± 6.7*	47.4 ± 5.7†	44.1 ± 4.6†	10.0 ± 0.2†
SH‐SY5Y‐L166P DJ‐1	100 ± 0	87.5 ± 6.6*	75.9 ± 7.9†	42.9 ± 4.5† ^,^ ‡	35.9 ± 3.6† ^,^ ‡	31.7 ± 5.4† ^,^ ‡	8.2 ± 0.5† ^,^ §
SH‐SY5Y‐C106S DJ‐1	100 ± 0	86.8 ± 6.9*	73.1 ± 1.4†	39.5 ± 0.6† ^,^ ‡	34.4 ± 2.2† ^,^ ‡	30.7 ± 5.7† ^,^ ‡	7.0 ± 0.9† ^,^ §

Note

Compared with control group: *p <* *0.05, ^†^ *p *<* *0.01.

Compared with SH‐SY5Y cells: ^‡^ *p *<* *0.05, ^§^ *p *<* *0.01.

### 3.5 *Cistanche* extracts inhibited H_2_O_2_‐induced reductions SH‐SY5Y cell viability

**Figure 4:**
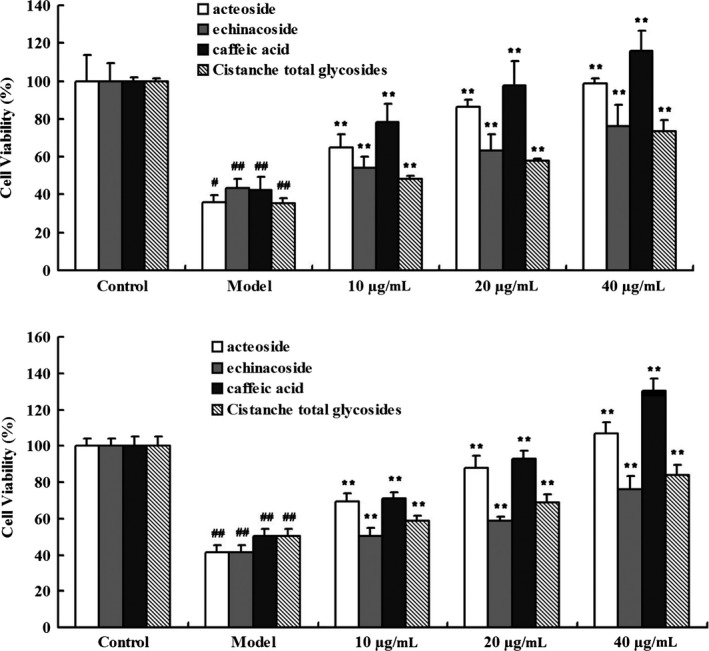
The effect of *Cistanche* extracts on cell viability in L166P (a) and C106S (b) DJ‐1‐transfected SH‐SY5Y cells treated with H_2_O_2_. ^#^ *p *<* *0.05, ^##^ *p *<* *0.01 vs. control; **p <* *0.01 vs. model

Figure 4 shows that the H_2_O_2_‐induced decrease in the viability of SH‐SY5Y cells transfected with L166P or C106S DJ‐1 was dose‐dependently inhibited by treatment with *Cistanche* extracts, including acteoside, echinacoside, caffeic acid, and *Cistanche* total glycosides (all 10, 20, and 40 μg/mL, respectively).

## 4 DISCUSSION

Mutations of *DJ‐1* are of being close association with autosomal recessive early‐occurrence PD (Bonifati et al., 2003; Park et al., 2015). In one Italian family, the cause is a missense mutation in *DJ‐1* (L166P) (Bonifati et al., 2003). Leu‐166 localizes in the middle of the C‐terminal helix (Bonifati et al., 2003). The presence of Pro, a strong helix breaker, likely destabilizes the terminal helix in the DJ‐1 mutant (Bonifati et al., 2003), leading to the unfolding of the C‐terminal portion of the protein, which would impair its homo‐oligomerization (Bonifati et al., 2003). In transfected cells, L166P DJ‐1 is unstable due to excessive degradation (Bonifati et al., 2003; Gorner et al., 2004; Moore, Zhang, Dawson, & Dawson, 2003). Bonifati et al. (2003) suggested that L166P mutation impairs the homodimerization and normal function of DJ‐1, and so leads to the early onset of PD.

In this study, we found that L166P DJ‐1 is present within a polymer/complex with a molecular mass of 70 kDa in transfected SH‐SY5Y cells. The structure of DJ‐1 was assumed to adopt the same α/β sandwich structure as protease PH1704 (Bonifati et al., 2003). Due to the similar structures of DJ‐1 and PH1704, we assumed that, like PH1704, DJ‐1 forms higher aggregates (trimers of dimmers). Gorner et al. (2004) showed that within transfected cells and the lymphocytes of PD patients, monomeric L166P DJ‐1 is present within the higher structures in transfected cells and lymphocytes of PD patients. This may be a direct result of L166P DJ‐1 protein misfolding or the formation of complexes between monomeric L166P DJ‐1 and other proteins. Moreover, in addition to the structure of the protein, L166P DJ‐1 mutation also changed the antioxidative function of DJ‐1 (Macedo et al., 2003; Taira et al., 2004). This is noteworthy, as oxidative stress is an important factor in PD, and SH‐SY5Y cells transfected with L166P DJ‐1 were more sensitive to H_2_O_2_ than untransfected cells. Combined with the previous research that wild type DJ‐1 transfected SH‐SY5Y cells was successfully established in our laboratory (Zhang, Wang, & Pu, 2007), we suggest that H_2_O_2_ induced L166P DJ‐1‐transfected SH‐SY5Y cells are a useful new cellular model of PD.

DJ‐1 has been reported to reduce oxidative stress (Taira et al., 2004; Zhou & Freed, 2005). One of the mechanisms is oxidation of the protein itself (Taira et al., 2004). Among the three Cys residues of DJ‐1, Cys‐106 is most sensitive to oxidative stress. Moreover, the antioxidative function of DJ‐1 is regulated through oxidation of Cys‐106 (Freed & Zhou, 2006), and mutation of Cys‐106 leads to loss of the antioxidative function of DJ‐1 (Blackinton et al., 2009; Kinumi, Kimata, Taira, Ariga, & Niki, 2004). DJ‐1 also acts as a molecular chaperone that inhibits formation of α‐synuclein aggregates (Shendelman et al., 2004) when Cys‐106 is oxidized to sulfinic acid (Zhou & Freed, 2005). These findings indicate that Cys‐106 is a key contributor to proper DJ‐1 function. As shown in Table 1, SH‐SY5Y cells transfected with C106S DJ‐1 were more sensitive to H_2_O_2_ than untransfected cells. Based on the previous research (Zhang et al., 2007), this also suggests H_2_O_2_ induced C106S DJ‐1‐transfected SH‐SY5Y cells may also be a useful cellular model of PD.

Using SH‐SY5Y cells transfected with L166P DJ‐1 or C106S DJ‐1, we detected the effect of *Cistanche* extracts and key bioactive compounds, including acteoside, echinacoside, caffeic acid, and *Cistanche* total glycosides on H_2_O_2_ induced reductions in cell viability. The present results show that acteoside, echinacoside, caffeic acid, and *Cistanche* total glycosides all increased cell viability in a concentration‐dependent manner, indicating a stable and linear relation between L166P DJ‐1 and C106S DJ‐1 levels and SH‐SY5Y cell viability.

In sum, we successfully established sensitive and stable H_2_O_2_ induced L166P DJ‐1‐ and C106S DJ‐1‐transfected SH‐SY5Y cell models of PD and confirmed that *Cistanche* extracts ameliorated the neurotoxicity induced by H_2_O_2_ in these two models. We anticipate that these two cellular models will be effectively used for PD research and *Cistanche* extracts may thus be useful for treating PD in the future.

## CONFLICTS OF INTEREST

The authors declare no conflicts of interest.

## AUTHOR CONTRIBUTIONS

CNA performed the experiment, XPP provided the funding, QW advised the molecular biological methods, and HNZ wrote the manuscript and supplied the publication costs.

## Data Availability

The data that support the findings of this study are available from the corresponding author upon reasonable request.
